# How the EU AI Act Seeks to Establish an Epistemic Environment of Trust

**DOI:** 10.1007/s41649-024-00304-6

**Published:** 2024-06-24

**Authors:** Calvin Wai-Loon Ho, Karel Caals

**Affiliations:** 1 Faculty of Law, Monash University, Melbourne, Australia; 2 PHG Foundation, University of Cambridge, Cambridge, UK; 3 Centre for Medical Ethics and Law, Faculties of Law and Medicine, The University of Hong Kong, Hong Kong, China; 4 Melbourne, Australia

**Keywords:** Trust, Trustworthiness, Artificial Intelligence, Regulatory sandbox, Real-world testing, Participatory Governance

## Abstract

With focus on the development and use of artificial intelligence (AI) systems in the digital health context, we consider the following questions: How does the European Union (EU) seek to facilitate the development and uptake of trustworthy AI systems through the AI Act? What does trustworthiness and trust mean in the AI Act, and how are they linked to some of the ongoing discussions of these terms in bioethics, law, and philosophy? What are the normative components of trustworthiness? And how do the requirements of the AI Act relate to these components? We first explain how the EU seeks to create an epistemic environment of trust through the AI Act to facilitate the development and uptake of trustworthy AI systems. The legislation establishes a governance regime that operates as a socio-epistemological infrastructure of trust which enables a performative framing of trust and trustworthiness. The degree of success that performative acts of trust and trustworthiness have achieved in realising the legislative goals may then be assessed in terms of statutorily defined proxies of trustworthiness. We show that to be trustworthy, these performative acts should be consistent with the ethical principles endorsed by the legislation; these principles are also manifested in at least four key features of the governance regime. However, specified proxies of trustworthiness are not expected to be adequate for applications of AI systems within a regulatory sandbox or in real-world testing. We explain why different proxies of trustworthiness for these applications may be regarded as ‘special’ trust domains and why the nature of trust should be understood as participatory.

## Introduction

With the approval of the proposed artificial intelligence act (AI Act) by the European Parliament (2024b), the European Union (EU) is set to be the first jurisdiction in the world to establish regulation on the use and supply of an AI system. The AI Act (Recitals 1 and 2)^1^ aims to boost innovation and employment, but also highlights the need to defend the principles of democracy and the rule of law, protect the environment, and ensure a consistent and high level of protection of health, safety, and fundamental rights that are enshrined in the EU Charter (European Union 2010). These conditions are considered necessary for the EU to become a leader in the uptake of trustworthy AI (Recital 2). In the light of these goals, it should not be surprising to find that the word ‘trustworthy’ and other related terms like ‘trust’, ‘trustful’ and ‘entrust’ appear more than 20 times in the legislation.

### The AI Act

Operationally, the AI Act adopts a risk-based approach whereby the extent of regulatory oversight of providers and deployers that put an AI system on the EU market, or where the output produced by their systems is used in the EU, will depend on the risk category to which that AI system is assigned. Although the protection of democracy, rule of law and environment is mentioned, the legal provisions are mainly concerned with risks to health, safety and fundamental rights. The way in which AI systems are constituted as regulatory objects reflects this risk-based approach. An AI system is defined as follows:

*A machine-based system that is designed to operate with varying levels of autonomy and that may exhibit adaptiveness after deployment and that, for explicit or implicit objectives, infers, from the input it receives, how to generate outputs such as predictions, content, recommendations, or decisions that can influence physical or virtual environments.* (Article 3, paragraph 1)

It includes a general-purpose AI (GPAI) system, which is defined as ‘an AI system which is based on a general-purpose AI model and which has the capability to serve a variety of purposes, both for direct use as well as for integration in other AI systems’ (Article 3, paragraph 66), with the term ‘general-purpose AI model’ being specified as:

*An AI model, including where such an AI model is trained with a large amount of data using self-supervision at scale, that displays significant generality and is capable of competently performing a wide range of distinct tasks regardless of the way the model is placed on the market and that can be integrated into a variety of downstream systems or applications, except AI models that are used for research, development or prototyping activities before they are placed on the market.* (Article 3, paragraph 63)

Simpler traditional software systems or programming approaches which are based on rules defined only by natural persons to automatically execute operations do not fall within the definition of an AI system. These simpler systems and approaches lack the capability to infer, or:

*The process of obtaining the outputs, such as predictions, content, recommendations, or decisions, which can influence physical and virtual environments and … to derive models or algorithms, or both, from inputs or data.* (Recital 12)

The capability to make inferences is derived from techniques that include machine learning approaches and logic- and knowledge-based approaches, and should go beyond basic data processing, enabled learning, reasoning or modelling. An AI system may operate independently or as a component of a product or system, with the potential to operate with varying degree of independence from human involvement, as well as adapt or change its functions through self-learning capabilities.

### Trust and Trustworthiness in the AI Act

With focus on the development and use of AI systems in the digital health context, we consider the following questions: How does the EU seek to facilitate the development and uptake of trustworthy AI systems through the AI Act? What does trustworthiness and trust mean in the AI Act, and how are they linked to some of the ongoing discussions of these terms in bioethics and philosophy? What are the normative components of trustworthiness? And how do the requirements of the AI Act relate to these components?

There are at least two readings of ‘trust’ and ‘trustworthiness’. A narrow reading considers trust and trustworthiness separately. By this reading, a person who trusts (or trustor) could choose to trust even though the trust recipient (or trustee) may not be trustworthy or that a trustworthy person may nevertheless be met with distrust. In the section that follows, we first consider asymmetric accounts of trust and trustworthiness that have emerged and how scholars in the fields of bioethics, law and philosophy have sought to address this epistemic imbalance and disconnect. We then explain why, in the context of digital health, we should adopt a broader view that considers trust and trustworthiness as constructs of social (and possibly institutional) epistemology. By our reading, the AI Act seeks to establish an epistemic environment of trust that links a trustor’s act of trusting with a trustee’s response to that act. The aims of these performative acts of cooperation are rendered symmetrical in relation to the external goals that connect the trustor to the trustee. For instance, Recital 3 of the AI Act indicates that a consistent and high level of protection throughout the EU is needed to achieve trustworthy AI. This is realised through the provision of legal certainty and through prevention of market fragmentation due to a differential application of national rules on the development, import and use of AI systems. From this vantage point, trust encompasses more than a belief, an emotion or a behavioural response, even though any one or all of them may be manifested in trusting. Within the epistemic environment of the AI Act, the trustworthiness of AI systems is a system-level concern with the assurance of uniform protection of public interests, individual rights and crucial socio-political norms in the deployment and use of these technologies in the EU (European Union 2012, Article 114). Hence, trust need not necessarily be linked to human cognition or relation, and trustworthiness is not limited to the presence of particular qualities or traits. It also follows that trust and trustworthiness need not be limited to human agents, but may apply to non-human ones as well. These arguments are set out in the section that follows.

We then examine the normative content of the governance regime that the AI Act establishes, which is also the socio-epistemological infrastructure of trust that controls and connects performative acts of trust through the hermeneutics of risk (Tuminello 2020; Kuran and Sunstein 1999). Under the AI Act, trustworthiness is based on performative requirements that serve as proxies of trustworthiness, to be explained below. These proxies seek to provide assurance that the use of an AI system will not result in harm to human health, safety or fundamental rights. In this sense, this infrastructure of trust operates as a governance mechanism for assigning proxies of trustworthiness and implements a division of epistemic labour among users, providers and deployers (whether current or prospective) of AI systems, as well as among EU member states and regulatory or governmental bodies like the AI Office and the AI Board (also discussed below). We consider four key features of this regime and the ethical principles that they seek to give expression to. As we will explain, the risk classification scheme is a core feature of this infrastructure of trust. Depending on the level and type of risks that are anticipated, the proxies of trustworthiness and the type of trust will differ accordingly. Taken in totality, it may be argued that this trust infrastructure does more than align values among different stakeholders in fostering the adoption of substantive norms of trust and trustworthiness vis-à-vis AI systems over time.

In the penultimate section of this paper, we discuss two innovative regulatory mechanisms referred to as a regulatory sandbox and real-world testing (RWT) of AI systems, and explain why we consider them as ‘special’ trust domains. We use the term ‘special’ to indicate specific proxies and mechanisms that operate as distinct regulatory spaces within the socio-epistemological infrastructure of trust. Importantly, the specification of these trust domains within the AI Act not only acknowledges the limitations of ‘standard’ proxies of trustworthiness that apply within the risk classificatory scheme, but also the need for new proxies of trustworthiness. The legislative provisions indicate that the infrastructure of trust may itself need to adapt and evolve through regulatory learning. In other words, innovation applies not only to AI systems but to the epistemic environment in which they operate. It is in relation to these special trust domains that we introduce participatory trust. Although substantive norms continue to apply, the nature of cooperation among stakeholders within a regulatory sandbox and in RWT is different in terms of cognitive demand, in relationality and in situational variability, among other conditions and considerations. It follows that a different type of trust emerges alongside new proxies of trustworthiness. We then conclude this paper with a brief explanation of how our propositions and arguments respond to the questions that are set out at the beginning.

## An Epistemic Environment of Trust

The topic of trust has drawn wide interest in disciplines including bioethics, philosophy and law, with more recent discussions extending to AI and other types of digital technologies. Our understanding of trust is multifaceted because different lines of disciplinary inquiry have provided a multitude of explanations as to its meaning (in conceptual and metaphysical analyses), its validity and justifications (in epistemic analyses) and its intrinsic value and legitimacy (in normative, political, social and psychological analyses), in a variety of contexts and in connection to different goals (McLeod 2023). Simply put, trust is a three-way relational cognitive construct around a trustor, a trustee and a matter that is being entrusted (Hardin 2002). Scholars have observed that trust and trustworthiness are not simply about reliability that is linked to competence and predictability (Lalumera 2024). For the trustor, certain conditions or motivations (linked to expectations, emotions and beliefs) are typically present for them to assume certain risks that the trustee may fail to deliver or meet. A decision to trust thereby renders the trustor vulnerable, as the decision could in some cases result in harm, be it physical injury or intangible ones like loss of self-respect and trauma from betrayal (Jones 1996; McLeod 2023). For the trustee, there must similarly be certain conditions or motivations to meet or deliver on what is entrusted. Scholars have identified these conditions or motivations to include goodwill, moral obligations and self-interest (Baier 1986; Hardin 2002; Nickel 2007), among others. However, a mismatch is apparent when one seeks to relate a one-place or dispositional analysis of trustworthiness with non-dispositional accounts of trust (Carter 2023). In other words, norms that apply to evaluate whether the trustor was right to trust need not be connected with those that determine if the trustee should have been deserving of trust. Yet trust and trustworthiness tends to be inseparable (Fricker 2007) when considering knowledge interactions that are linked to power dynamics (e.g. epistemic injustice). Trust operates as a continuum rather than a binary, and many of its challenges tend to relate to trustworthiness—at least where trust involves some level of cognition. For this reason, Onora O’Neill (2002; 2018) has famously argued that inquiring into trust is not fruitful unless it is linked to trustworthiness. The focus would then be on evidence of honesty, competence and reliability, so that a trustor is then able to decide if a trustee is worth the trust or not. Rather than to analyse trust in terms of trustworthiness, Carter (2023) proposes to evaluate the latter like the former in terms of a state, an action or a process that has a constitutive aim (Carter 2022; Sosa 2021). Both trust and trustworthiness encompass performance that pertains to a cooperative exchange or engagement assessible in terms of varying degrees of success. By this ‘trust as performance’ approach, evaluative norms that regulate attempts, dispositions and achievements would apply symmetrically to trust and trustworthiness. As Carter observes (2023, 385): ‘In the good case where cooperation is working as it should, the trustor matches her achievement in trusting with the trustee’s achievement in responding to trust. In both of these achievements (of apt trust and apt reciprocity) competence is manifested in success.’ There are a number of advantages to the introduction of symmetric evaluative normativity between a trustor and a trustee, which includes a proposition that trust may be extended to non-human agent.

### Trust beyond Humans

More recent arguments have been made to extend trust and trustworthiness beyond human agents to include non-human entities like institutions (Townley and Garfield 2013; Oreskes 2019), governments (Hardin 2002; Budnik 2018) and robots (Coeckelbergh 2012; Sullins 2020). The jury is still out as to whether trust and trustworthiness may apply to these non-human entities (like AI systems) since institutions have goals and devices have functions, which differ from human intentions and motivations. By this view, trust and trustworthiness are to be confined to interpersonal relationships, whereas reliability is what humans really expect of non-human entities (Hawley 2014). It is beyond the scope of this paper to engage fully with this issue, but we only mention here that plausible arguments have been made that trusting relationship could be extended to non-human entities like organisations (as internally complex groups) (Bennett 2024) and institutions (Gallagher and Petracca 2024), while still sustaining the distinction between trust and reliability.

One such line of argumentation applies to the idea of the socially extended mind or cognitive institutions (Gallagher 2018; Slors 2019) to show that institutions like legal systems and capital markets may be trustworthy and thereby deserving of trust. The basic idea, as Gallagher and Petracca (2024) explain, is that cognitive institutions (e.g. science, law, healthcare and economics) enable those who engage with them to draw on cognitive processes to address specific problems or achieve certain types of cognitive accomplishments. Intersubjective interaction and task dependency have crucial roles in these institutions, which operate as sites of social practices with normative expectations and constraints, and that involve responsibility for carrying out particular tasks. If we apply this approach to a healthcare setting, it is not difficult to see that hospital administrators, nurses, pharmacists, doctors, therapists, medical social workers and other hospital staff are responsible for tasks that are interdependent and interlinked as a whole. Typically, healthcare facilities have clear mechanisms, procedures and protocols to ensure that what nurses do may be understood by referring to what the tasks of doctors and pharmacists are. If, through due diligence, we find these mechanisms, procedures, and protocols to be up-to-date and comprehensive, we may assess the facility to be reliable for having covered all anticipated care needs and concerns. Yet one may still not trust this healthcare facility if we do not think that its healthcare staff genuinely care about patients, or that these mechanisms, procedures and protocols will be faithfully applied. So, when we say that we trust a healthcare facility or that the facility is trustworthy, we do intend to include a non-human entity in a trusting relationship, and we do not mean that it is merely reliable without more. How about other non-human agents like an AI system?

### e-Trust

In the context of digital health, Primiero and Taddeo (2012) explain that trust and trustworthiness have a transversal character in that they occur among human agents and non-human agents and could extend beyond human–human interactions, to include human–non-human and non-human–non-human interactions. Additionally, the basis of trust is not necessarily cognitive in an evaluative sense since a potential trustor cannot be expected to carefully assess the trustworthiness of a trustee all the time. If this were to be the case, trust would be an endemic problem since no trustor will have the resources to carry out intensive evaluations for every entrustment decision, assuming that this can always be done. However, trust would typically be exercised under certain conditions that indicate a level of trustworthiness, as we will shortly explain. These conditions constitute what some scholars have termed ‘e-Trust’ to highlight the role and impact of artificial/non-human agents in distributed systems where interactions are intermediated by digital devices and in the absence of any physical or direct human contact (Taddeo 2009; Taddeo and Floridi 2011). Within this e-Trust environment (now a recognised lexicon in the Cambridge Dictionary), a variety of digital devices are entrusted with the distribution of tasks within society without requiring its members to be overburdened with supervision or control. The extent of trust that one environment requires in order to work well will differ from another, but it is the facilitative (rather than affirmative) effect of trust and trustworthiness that is emphasised. In other words, trust and trustworthiness become themselves indicators of the quality and effectiveness of the interactions between human and non-human agents in achieving particular goals within that environment. We consider this line of reasoning to be similar to Carter’s (2022) focus on cooperative exchange in his ‘trust as performance’ approach. The norms of trust and trustworthiness apply more broadly to the digital environment in the evaluation of the extent to which constituent human and non-human agents could collectively deliver the outcomes to which they are entrusted.

In the e-Trust context, the ‘incremental model of trust’ put forward by Andrea Ferrario et al. (2020) reflects this evaluative approach, but introduces risk evaluation that is implicit in the element of control. The first of this three-layer model of trust is a non-cognitive account of trust that is based on the concept of reliance. Referred to as simple trust, the trustor relies on the trustee to perform an action to pursue a goal within a specific context without intentionally seeking further information about the trustee’s capabilities to achieve that goal. Simple trust is thereby a non-cognitive account of trust and is said to arise from contingent causes (e.g. lack of space, resource, time or capacity) and structural ones (e.g. young children instinctively trust their parents). If the trustor is able to entrust an action to a trustee with some degree of confidence (through subjective assessment based on past experience, for instance) that the intended goal will be achieved in a particular context, reflective trust is said to arise. Should the trustor decide to exercise some degree of control over the trustee despite the confidence that the trustor has, deviant reflective trust is said to be manifested. The final layer in this model is paradigmatic trust, where there are sound epistemic and pragmatic reasons for the trustor to entrust an activity with the trustee. These reasons may be objective (as facts) or subjective (as belief), but they relate ultimately to the outcomes that are expected of that particular digital context and environment.

Our interest in this model is the hermeneutics of risks implicit in the model and underscores the scalar (or ‘incremental’) relationship between a basis of trust and the degree of control. By linking the parameters of cognition (i.e. from non-cognitive reliance to cognitive judgment of trustworthiness) and control within a cooperative environment, this approach better accounts for the different degrees of understandability and intuitiveness that AI systems could exhibit. In principle, a fully adaptive AI system will always lack trustworthiness because it is inscrutable and nonintuitive (Selbst and Barocas 2018) and hence fall short of ethical requirements. In an environment of trust however, the requirement of explainability is but one condition of trustworthiness, and other engendering conditions (which we explain in the section that follows) could well provide justifications as to why such an AI system may be entrusted with a specific goal. Trust then has a more dynamic character that changes over time through interactive actions and mechanisms that include negotiation and coordination. As Taddeo observes (2017, 566):

*In the medium- and long-term, too little trust may affect the internal dynamics of the system and limit its development; but too much trust may lead to the collapse of a system if it results in a lack of control and coordination. Striking the right level of trust it is a delicate matter and requires considering the system (mature information societies…), the expectations of the trustor (the tasks that human agents delegate), and the nature of the trustee (e.g. the digital technologies)*.

### Social Epistemology of Trust

We find a parallel line of reasoning in studies on public trust in the social epistemology of science, which posits that there is a social dimension to all aspects of science, whether it is in the production of scientific knowledge or its reception, transmission and uptake by the rest of society. For instance, Contessa (2023) proposes that individual trust in science is the result of a habit that is neither voluntary nor fully rational, and this is largely the result of the attitudes towards science held by those in that individual’s social and epistemic network. To address the problem of distrust, our attention should be directed at social and structural changes, rather than interventions at the individual level. Adapting Contessa’s social approach to trust relationships, public trust in an institution like healthcare (and by extension, digital health) is essentially a form of social trust that is concerned with an efficient division of epistemic labour between the different human and non-human agents that subsist in the socio-epistemic infrastructure of that society. Public trust reflects the quality of the epistemic environment of trust, or what Annette Baier (1986, 234) describes as ‘a climate of trust’ that we inhabit. A sound epistemic environment is not to be taken for granted as it can become polluted or corrupted when imbued with features that allow our cognitive vulnerabilities and weaknesses to be exploited. Nguyen (2023) labels such an environmental phenomenon as ‘hostile epistemology’, with features that could include other people, communities, cultural practices, institutional structures and technologies. As he argues, hostility does not require intentionality and is hence not necessarily linked to epistemic vice. Instead, it reflects the human condition and our inherent cognitive limitations that necessitate our reliance on heuristics (or mental shortcuts) and on trust.

## A Governance Regime as Socio-epistemological Infrastructure of Trust

We consider a key goal of the AI Act to be the establishment of a socio-epistemological infrastructure to nurture and sustain a trust-promoting epistemology and environment that is applicable to AI systems in the context of digital health. This infrastructure takes the form of a governance regime that is focused on alleviating or mitigating specific harms that can arise from trusting relationships that involve an AI system. The normative work on a trustworthy AI environment in the EU has been done by the independent High-Level Expert Group on AI (AI HLEG) appointed by the European Commission (2019) in its *Ethics Guidelines for Trustworthy AI*. The seven ethical principles identified by the AI HLEG are explicitly mentioned in the AI Act (Recitals 7 and 27) as: (1) human agency and oversight; (2) technical robustness and safety; (3) privacy and governance; (4) transparency; (5) diversity, non-discrimination and fairness; (6) social and environmental well-being; and (7) accountability. Adopting an essentially risk-based approach however, there will inevitably be uneven treatment of these principles. For this reason, the AI Act does not preclude the application of other governance or regulatory regimes. Additionally, the regime seeks to give effect to other normative commitments that are mainly based on core legal principles and human rights. AI systems must operate in accordance to the values of respect for human dignity, freedom, democracy, equality, the rule of law and respect of human rights, which are laid down in Article 2 of the Treaty on European Union (Recital 6). Broadly speaking, these requirements seek to ensure that AI systems are designed, developed and used as a human-centric technology and as a means to increase human well-being. Obligations that follow from this include the introduction of measures to ensure AI literacy among all relevant actors in the AI value chain and to introduce follow-up actions (Recital 20). It is beyond the scope of this paper to engage with the legal and human rights implications of the AI Act, but one of us (Ho 2023) has considered the relationship between human rights and AI systems that operate as medical devices in the context of the EU and elsewhere. Here, we show how four key features of the regime introduced by the AI Act seek to give effect to the ethical principles put forward by the AI HLEG with the overarching goal of establishing a trustworthy environment for AI development and implementation.

### Oversight

The governance regime established under the AI Act has a number of distinctive and overlapping features, of which we focus on four of them to illustrate what the EU considers to be the conditions of a trustworthy AI environment. First, it sets out the key responsibilities of the European AI Office, which was established by the European Commission (EC) as part of the administrative structure of the Directorate-General for Communication Networks, Content and Technology, and hence not intended to affect the powers and competences of EU member states or the bodies, offices and agencies of the EU in the supervision of AI systems (European Commission 2024a). The roles and responsibilities of the AI Office are many and crucial to ensure that the ethical principle of human agency and oversight is observed. This principle requires AI systems to be developed and used as a tool that serves people, respects human dignity and personal autonomy and functions in a way that can be appropriately controlled and overseen by humans. Tasks assigned to the AI Office that reflect this principle include the following:

- Developing a template for a questionnaire in order to facilitate compliance with the requirement to conduct a fundamental rights impact assessment (where applicable) and to reduce the administrative burden for deployers (Recital 96)
- Providing a template to enable a provider to set out as a summary and in narrative form data that is used in the pre-training and training of GPAI models (Recital 107)
- Monitoring whether the provider has fulfilled obligations without verifying or proceeding to a work-by-work assessment of the training data in terms of the copyright compliance (Recital 108)
- Establishing a methodology for the classification of GPAI models with systemic risks, along with thresholds, benchmarks and indicators for assessing high-impact capabilities after engaging with the scientific community, industry, civil society and other experts (Recital 111)
- Providing coordination support for joint investigations that may need to be conducted by an EU member state’s regulatory agency (referred to as market surveillance authority or ‘MSA’) and by the EC for specific high-risk AI systems (Recital 160)
- Providing support to regulatory agency that is unable to access certain information related to the GPAI model in investigation (Recitals 161 and 163)

Many of these tasks are also relevant to the principle of transparency, which requires AI systems to be developed and used in a way that allows appropriate traceability and explainability, while making humans aware that they communicate or interact with an AI system, as well as duly informing deployers of the capabilities and limitations of that AI system, and the rights of affected persons.

### Collaborative Governance

The second feature of the AI Act is distinctive for its collaborative approach on many different fronts and in relation to varied stakeholders along the AI value chain. It appears to us that the governance arrangements reflect the principles of accountability and the triplex principles of diversity, non-discrimination and fairness. As the AI HLEG explains, accountability requires the design and use of AI systems, and applicable codes of conduct and best practices, to be consistent with the ethical principles and auditable. A diverse range of stakeholders should be involved to promote equal access, gender equality and cultural diversity, while avoiding discriminatory impacts and unfair biases. A reason for adopting a definition of AI systems that is closely aligned with that of the OECD’s is to facilitate international collaboration (Recital 12). Regulatory agencies of EU member states and EU agencies (particularly the AI Office) are encouraged to build and utilise centralised EU expertise and synergies (Recital 162), to forge strong links with the scientific community in order to support its work and to involve a wide range of stakeholders. To this effect, the European AI Board is established by Article 65 to facilitate a smooth, effective and harmonised implementation of the AI Act in a manner that ‘reflect[s] the various interests of the AI eco-system and [is] composed of representatives of the Member States’ (Recital 149). The AI Board is responsible for a number of advisory tasks (including contributing to guidance on matters related to the implementation of the AI Act) and is to provide advice to the EC and the member states (and their national competent authorities) on specific questions relating to AI. The AI Board is also tasked with providing a platform for cooperation and exchange among national regulatory agencies (or MSA) and notifying authorities and to cooperate with relevant EU bodies, expert groups and networks, especially those that operate under EU regulations on data, digital products and services, among other responsibilities (Article 66).

The AI Act envisages a wider pool of stakeholders to support the work of the AI Board and the EC through a scientific panel of independent experts and an advisory forum. One task of the AI Board that is likely to require wide stakeholders’ input is in supporting the EC to promote AI literacy, public awareness and understanding of the benefits, risks, safeguards and rights and obligations in relation to the use of AI systems (Article 66(f)). The scientific panel of independent experts provides technical advice and input to the AI Office and to national regulatory agencies and is able to launch qualified alerts of possible risks to the AI Office (Articles 66(n) and 66(o)). Additionally, an advisory forum, which is to reflect a balanced selection of stakeholders from various sectors including industry, start-ups, civil society and academia, is expected to provide inputs to the AI Board and to the EC (Article 67). To ensure that concerns with health, safety and fundamental rights are adequately addressed, the AI Act requires that the Fundamental Rights Agency, European Union Agency for Cybersecurity, the European Committee for Standardization, the European Committee for Electrotechnical Standardization and the European Telecommunications Standards Institute are involved as permanent members of the advisory forum (Article 67(5) and Recital 150). Arrangements relating to the scientific panel give expression to the principle of technical robustness and safety, whereas those concerning the advisory forum draw together the principles of technical robustness and safety and the triplex principles of diversity, non-discrimination and fairness. The wide interpretation of robustness as encompassing resilience against attempts to alter the use or performance of AI systems lends support to reading these two principles together.

### Risk Anticipation and Management

Third, a substantial part of the governance regime is directed at high-risk AI systems, with focus on their intended purposes, as well as the generally acknowledged state of the art of the AI and AI-related technologies (Article 8(1)). A key feature that sets a high-risk AI system apart from a prohibited AI system is the viability of a risk management system that could be established, implemented, documented and maintained in relation to the former (Article 9(1)) but not the latter. It is thereby a governance arrangement that prioritises the principle of social and environmental well-being, as the long-term impact of AI systems on the individual, society and democracy must be carefully monitored and assessed. It follows that the risk management system should be ‘a continuous iterative process planned and run throughout the entire lifecycle of a high-risk AI system’ that allows for ‘regular systematic review and updating’ (Article 9(2)). The principles of oversight, technical robustness, transparency and data governance make crucial supportive contributions to the proper functioning of risk management systems.

The types of ‘risk’ in these AI systems are therefore those that may be reasonably mitigated or eliminated through the development or design or through provision of adequate technical information (Article 9(3)). In this respect, detailed information is to be provided in technical documentation concerning the monitoring, functioning and control of the AI system, particularly in relation to its capabilities and limitations in performance (with specific reference to the degrees of accuracy for specific persons or groups of person on which the system is intended to be used and the overall expected level of accuracy in relation to its intended purpose); the foreseeable unintended outcomes and sources of risks to health and safety, fundamental rights and discrimination in view of the intended purpose of the AI system; and the human oversight measures that are put in place in accordance with Article 14, including technical measures to facilitate the interpretation of the outputs of AI systems by the deployers and specifications on input data (Article 11(1); Annex IV, paragraph 3). Data quality criteria must also be observed, along with appropriate data governance and management practices based on the intended purpose of the AI system (Article 10). For instance, datasets used for training, validation and testing must be relevant, sufficiently representative and, as far as possible, free of errors and complete in view of the intended purpose (Article 10(3)). Along with mechanisms to ensure human-centredness, transparency, accuracy, robustness and cybersecurity, these are but a few regulatory tools introduced under the AI Act to manage or avoid risks of harm to health, safety and human rights.

### Pro-innovation

Finally, the AI Act is to be pro-innovation in its prescription of the proxies of trustworthiness (further discussed below) and in the division of regulatory responsibilities. While regulatory control should ensure that specified harms are avoided or mitigated through appropriate management, trustworthiness of AI systems is ultimately aimed at creating an environment of trust to facilitate AI development and uptake. In keeping with the principle of accountability, member states are to ‘take all necessary measures to ensure that the provisions of … [the AI Act] are implemented, including by laying down effective, proportionate and dissuasive penalties for their infringement, and in respect of the ne bis in idem principle’ (Recital 168 and Article 99). It proposes that the upper limits for setting the administrative fines for specific infringements be clearly set out and that all relevant circumstances of the specific situation be taken into account, including the size of the provider. Normative commitments are also applicable to regulatory agencies, and these include the need to respect confidentiality of information and data obtained in carrying out regulatory tasks and to protect intellectual property rights (IPR), public and national security interests, as well as some context specific requirements like integrity of criminal or administrative proceedings and the integrity of classified information. These commitments are indicated as necessary to ensure ‘trustful and constructive cooperation’ of authorities at the EU and national level (Recital 167 and Article 99).

The AI Act explicitly states that it should support innovation, respect freedom of science and should not undermine research and development (R&D) activity (Recital 25 and Article 2). For this reason, the legislation will not apply if an AI system is being developed or tested within a clinical trial context and is thereby not being put into service or placed on the market. An AI system that is used for military, defence or national security purposes is similarly excluded from the AI Act. On a similar rationale, an AI system that is expected to advance public interest may be developed by using personal data collected for other purposes, subject to specified conditions and safeguards to prevent or mitigate significant risks to safety, health and fundamental rights that may arise during the development, testing and experimentation in the sandbox (Recital 140), which is a particular domain of trust that we discuss below.

There are plentiful cautions against stifling innovation through over-regulation due to overlapping regulatory competencies of regulatory agencies. Afterall, the faithful observance of normative requirements could detract a trustee from the intended goal. For instance, transparency requirements are generally important, but they are not foolproof and could be counterproductive. When mechanisms are introduced to allow for the performance of an institution to be assessed for satisfaction, they could instead incentivise dishonesty (O’Neill 2002). Or where these requirements are introduced to render expert knowledge publicly accessible, experts may then be forced to lie, oversimplify or limit their actions to those that are explicable to non-expert since the very nature of their expertise is its inaccessibility to non-experts (Nguyen 2022). Various arrangements are set out in the AI Act to avoid overregulation through the division of regulatory responsibility. A regulatory agency appointed by an EU member, along with a notifying authority, may be assumed to have the independence to exercise its powers. These include effective investigative and corrective powers, the related power of obtaining access to all personal data that are being processed as well as all information necessary for the performance of its tasks (Recital 159 and Article 57(11)). However, for an AI system that is based on a GPAI model and where the model and AI system are provided by the same provider, the AI Act states that supervision should take place at the EU level through the AI Office. In all other cases, national regulatory agencies remain responsible for the supervision of AI systems (Recital 161). And if a GPAI system may be used directly by developers for at least one purpose that is classified as high-risk, the national regulatory agency concerned should cooperate with the AI Office to carry out evaluation of compliance and to inform the AI Board and other national regulatory agencies of the outcome. Where the latter should request for assistance from the AI Office to investigate a high-risk AI system, the procedure regarding mutual assistance in cross-border cases in Chapter VI of Regulation (EU) 2019/1020 applies (Article 75(3)).

As we have discussed, trustworthy AI is an ‘infrastructural’ project that seeks to create a socio-epistemic environment of trust through a system of governance. Within this system, an AI system is constituted as ‘risk object’ so that it may be prohibited (for posing unacceptable risks), controlled through risk management (if deemed to be high risk) or assessible after certain conditions are met (if low risk). At least for this governance regime and in respect of AI systems that are not prohibited, risk is the basis for devising, moderating and assigning proxies of trustworthiness. As a practical matter, an epistemic division of labour is achieved since reliability in the sense of the AI HLEG’s principle of technical robustness, and safety becomes part of the trust continuum. However, the fact that a trustor is able to rely on a low-risk AI system to perform a task within a particular context is not reliance simpliciter, as an assurance of trustworthiness is provided through certification by the provider. High-risk AI systems bear the *conformité européenne* (“CE”) marking (which may be physical and/or digital) to indicate their conformity with the AI Act in order to move freely within the EU market (Recital 129, Article 16(h), Article 23(1)(c), Article 24, Article 48 and Article 83). As a form of heuristic, the accuracy and reliability of the CE certification process and conditions are subject to regulatory (i.e. human) oversight. To be sure, we do not read the AI Act as conflating trustworthiness with the acceptability of risks (Laux et al. 2024), but rather as applying the latter as a means to better align AI systems and regulation (Díaz-Rodríguez et al. 2023). As we have indicated above, we read the AI Act as an attempt to establish an epistemic environment of trust through legislative means, rather than to engineer trust. Our goal here is not to examine the complex relationship between trust and risk, but only to highlight that different evaluation and perception of risks necessitate different proxies of trustworthiness, notably through the intermediation of regulatory authorities. These different proxies of trustworthiness could be interpreted as being linked to different types of trust (Nickel and Vaesen 2012; Siegrist 2021; Solberg et al. 2022), such as participatory trust discussed below. We first consider how the AI Act has applied a regulatory construction of risk in structuring the epistemic environment of trust.

## Structuring the Epistemic Environment of Trust

In the previous sections, we have explained how the AI Act seeks to establish an epistemic environment of trust. A central feature of this environment is a governance regime that serves as a socio-epistemic infrastructure that relates trustor and trustee (and their cognates of trust and trustworthiness) to legally sanctioned social goals that are premised on normative values and principles articulated by the AI HLEG. Operating within this infrastructure, trust and trustworthiness encompass performative acts that are directed towards specific aims. As these aims are defined within the governance regime, performative acts of trust and trustworthiness may be assessed based on the extent that they have been successful in realising the overarching goals set out in Article 1 of the AI Act. Consistent with Carter’s (2022) performance normativity, performative acts may also be assessed as to their degree of competence and aptness, given that technical and normative prescriptions are intrinsic to the legislative goals. Acts that succeed in promoting innovation or uptake of an AI system may nevertheless fall short of trust and trustworthiness if they should lack competence or aptness. In this respect, we have considered how the governance regime seeks to give effect to specific normative principles, while remaining open to the application of other normative concerns, such as human rights prescriptions. In a paper that addresses similar concerns of trust and trustworthiness in human health research, Harvey and Laurie (2024, 7) provide this eminently lucid and instructive observation:

*…if the performance of trust is oriented towards the system itself, and if that system and its values root out untrustworthy performances and hold the actors accountable, then that trust is arguably not misplaced. Bad apples do not necessarily rot the barrel.*

As they demonstrate, the performative framing of trust and trustworthiness provides a basis to understand and evaluate successful cooperative exchange in human health research. In that context, success arises in the form of realising the social value from research, but it is not social value at any cost since assessment of the research will include competence of researchers as well as its aptness based on ethical and governance considerations and requirements. By linking the act of trusting (e.g. through participation in research) and the act of seeking to demonstrate trustworthiness (e.g. by a researcher through clearly articulating and implementing commitments set out in a research protocol) as performances directed towards a common goal, trust and trustworthiness are placed on a levelled ontological plane that in turn enables normative evaluation. Further instructive in their analytical approach is the construct of ‘proxies of trustworthiness’, which are ‘the operational tools used to perform trustworthiness’ (Harvey and Laurie 2024, 6). Performative acts that pertain to consent, anonymization, public engagement, openness and accountability are illustrative of common proxies of trustworthiness in that context which, taken together, form a character profile of trust-seeking behaviour.

In the section that follows, we first consider how the language of risk is instrumental in linking competence to aptness of performative acts within the governance regime of the AI Act. These performances relate to the categorisation, management and mitigation of risks and thereby serve as proxies of trustworthiness that providers and deployers of AI systems use to perform trustworthiness. There are however two types of application of AI systems where these proxies may not be adequate. The AI Act demarcates the application of these AI systems contextually as occurring within a regulatory sandbox and in real-world settings. Referring back to the analytical framework of Harvey and Laurie (2024), we consider their discussion on ‘risks to proxies of trustworthiness’ to be especially pertinent. They explain that there may be a need to re-evaluate the values and proxies and to consider if new proxies of trustworthiness are required where there is a material change in circumstances, for instance. We refer to the differential treatment of AI systems applied in a regulatory sandbox and in a real-world setting as ‘special trust domains’ because different performative acts are required. This is implicit in the organisation of the AI Act itself, where the regulatory sandbox and real-world testing are grouped together under Chapter VI, which is entitled ‘Measures in Support of Innovation’. In these domains, the performative acts of the trustor and trustee, and indeed in their cooperation with national and EU-level authorities, appear to assume a qualitatively different character, giving rise to what we term as ‘participatory trust’. The nature of participatory trust and its relevance to these special trust domains are discussed in the penultimate section of the paper.

### Trustworthiness through Risk Evaluation in an Environment of Trust

The way an AI system is constituted under the AI Act as a regulatory object makes clear that regulatory attention and resources are to be directed at preventing or alleviating potential harms to human health, safety and fundamental rights in order to sustain trust. For this reason, a wide range of specific AI practices (i.e. the act of placing on the market, putting into service, or using an AI system) are prohibited on the basis of their harmful impact. The following AI systems are some of the prohibited AI practices that are pertinent to the healthcare and digital health contexts (Article 5):

(i) AI systems using subliminal, manipulative, or deceptive techniques to distort people’s or a group of people’s behaviour and impair informed decision-making, leading to significant harm
(ii) AI systems exploiting vulnerabilities due to age, disability or social or economic situations, causing significant harm
(iii) Biometric categorisation systems inferring race, political opinions, trade union membership, religious or philosophical beliefs, sex life or sexual orientation (unless for lawful labelling or law enforcement purposes)
(iv) AI systems inferring emotions in workplaces or educational institutions, unless for medical or safety reasons

For AI systems that are not prohibited, classification rules in the AI Act designate an AI system into one of three risk categories, whereby the degree of regulation and assigned proxies of trustworthiness apply in proportion to the risk of harm. The term ‘risk’ is defined as the combination of the probability of an occurrence of harm and the severity of that harm (Article 3(2)) that is posed to human health, safety and fundamental rights, although the overarching purpose of the AI Act is stated more broadly as improving ‘the functioning of the internal market and promoting the uptake of human centric and trustworthy artificial intelligence (AI), while ensuring a high level of protection of health, safety, fundamental rights enshrined in the Charter…and supporting innovation’ (Article 1(1)). Risk classification is based on the intended purpose of the AI system, its functions and modalities, taking into account the nature and amount of data processed and used by the AI system, the extent to which the AI system acts autonomously and the possibility for a human to override a decision or recommendation that may lead to potential harm, power imbalance, the magnitude and likelihood of benefit from the deployment of the AI system and other considerations set out in Recital 52 and Article 7(2).

Under this risk classification scheme, the most demanding proxies of trustworthiness apply to an AI system that is of ‘high risk’ and is subject to the greatest degree of regulatory scrutiny. A high-risk AI system may be a safety component of a medical device regulated under the *Medical Devices Regulation* (European Parliament and European Council 2017) or other specific regulatory regimes (e.g. *General Data Protection Regulation* (GDPR) of the European Parliament and European Council (2016)) or used in specific areas listed in Annex III of the AI Act, which includes AI systems that impact on access to healthcare services and life and health insurance. Even if an AI system does not pose a significant risk of harm to health or safety, it will be classified as high-risk if the AI system performs profiling of natural persons (Recital 10, Article 6(2) and paragraphs 6(d) and 6(e) of Annex III) owing to concerns about the potential violation of fundamental rights. All providers of a high-risk AI system must run a conformity assessment procedure (based on internal controls or with a notified body) for it to be sold or used in the EU. Conformity requirements include testing, data training and cybersecurity, post-market monitoring and remedial action plans. In some cases, a fundamental rights impact assessment will need to be conducted (Article 27). Where providers are bodies governed by public law or private actors providing public services, and for deployers that are banking and insurance service providers using AI systems listed as ‘high-risk’, a fundamental rights impact assessment should enable the ‘deployer to identify the specific risks to the rights of individuals or groups of individuals likely to be affected, and identify measures to be taken in case of the materialisation of these risks’ (Recital 96).

Read in relation to the key features of the regulatory regime that we discussed above legislative provisions of the AI Act that are directed at high-risk AI systems attempt to give effect to the ethical principles of the AI HLEG in differing orders of priority. Risk management and alleviation through means that include the establishment of a risk management system (Article 9) relate to the principle of human agency and oversight, the principle of technical robustness and safety and the principle of social and environmental wellbeing. The requirement to put in place appropriate data governance and management practices (Article 10) is primarily concerned with the principle of privacy and data governance, whereas the requirement to ensure traceability through record-keeping (Article 12) is premised on the principles of transparency and accountability. Providers are to supply information on AI systems to deployers and regulators to ensure transparency (Article 13), while incorporating human oversight (Article 14). Assurance of an appropriate level of accuracy, robustness and cybersecurity through design and development (Article 15) must also be provided to ensure diversity, non-discrimination and fairness. Here too, the AI Act cautions against over-regulation and the AI Office has been specifically tasked to address this concern.

Where an AI system is not of high-risk but presents risks associated with lack of transparency in its use (for instance, in enabling impersonation, manipulation or deception), regulatory requirements relating to transparency and information apply (Articles 13 and 50). It is not clear from the AI Act if such an AI system constitutes a risk category that is distinct from the binary of ‘high-risk’ and ‘not high-risk’ in the risk classificatory scheme, although explanatory materials appear to recognise an intermediary category of ‘limited risk’ (European Commission 2024b) or ‘transparency risk’ (European Parliament 2024a). As illustration, the European Commission indicates that if an AI system is used as chatbot, the provider must ensure that humans are aware that they are interacting with a machine so they can take an informed decision to continue or step back. Additionally, the provider must ensure that AI-generated content is identifiable so that the public is aware that published text on matters of public interest is artificially generated or not. This requirement also applies to audio and video content constituting deep fakes.

An AI system that does not profile natural persons is considered not to pose a significant risk of harm if it is intended to perform a narrow procedural task, improve the result of a previously completed human activity and detect decision-making patterns or deviations from prior decision-making patterns and is not meant to replace or influence the previously completed human assessment without proper human review or perform a preparatory task to an assessment relevant for the purpose of the use cases listed in Annex III (Article 6(3)). A provider of such an AI system only needs to document its assessment before the system is placed on the market or put into service and is subject to registration obligation (Article 6(4)).

Trustworthiness as a matter of performative requirements is most demanding for high-risk AI systems, as well as those that could violate fundamental rights of individuals owing to the lack of transparency, within this epistemic environment of trust of the AI Act. Circling back to the model proposed by Ferrario et al. (2020), reflective trust and its deviant form find application in relation to these AI systems. In contrast, simple trust may be considered to apply to low-risk AI systems, where ‘standard’ proxies of trustworthiness provide adequate assurance that there will be no significant risks of harm to health, safety or fundamental rights associated with their use. This trust extends to regulators at the national and EU levels, as they are entrusted with the responsibility of setting standards and specifying requirements that are up-to-date and robust. If deployers and users so wish, they have the option of seeking more information to support their belief or expectation. Where such an option is exercised, the nature of trust may perhaps be better understood as a paradigmatic (rather than simple) one.

What this model of trust does not appear to speak to is a participatory form of trust where there are reasons to question the adequacy of existing proxies of trustworthiness or when epistemic division of labour in the use of CE markings could not be properly applied. Instead, participatory trust requires different degrees of negotiation over time and potentially across overlapping contexts. We find this participatory form of trust to be applicable in a distinct epistemic environment that the AI Act establishes for testing of AI systems within a regulatory sandbox or in a real-world setting.

## Participatory Trust in Trust Domains

Perhaps the most innovative feature of the AI Act is in the creation of a distinct environment to establish and calibrate new proxies of trustworthiness for (mainly high-risk) AI systems applied in two ways or settings. This environment shares some similarities with the more established regulatory regimes of clinical trial regulation and associated ethical governance of research involving human participants, particularly requirements relating to consent and related arrangements. There are important differences among these different regimes, and we have discussed them elsewhere (Ho and Caals 2021; Ho 2021). For present purposes, we focus on how the AI Act constitutes a regulatory sandbox and real-world testing (RWT) as mutually non-exclusive domains of application that are distinct from the risk-based infrastructure that sustains an epistemic division of labour. These domains are more anticipatory in character and seek to encourage innovation in AI systems through means that include regulatory innovation. In other words, the socio-epistemic infrastructure is itself subject to change as regulators and regulatory systems learn, adapt and evolve.

Drawing broadly from Stewart’s (2024) work on trust domains, which she defines as sets of expected behaviour or attitudes held by individual trusters. Since trust domains consist of nested sets of expectations, breaking of trust may arise due to misunderstanding, mismatch or even direct conflict of expectations between the trustor and the trustee, rather than through negligence, incompetence or ill will. As she explains, the trust domains seek to enable trustor and trustee to determine the scope of trusting relationship, and the rigidity, as well as ordering of these expectations. Whereas Stewart treats trust domains as individualised phenomena rather than social epistemology, we think that this concept can find wider application while retaining a performative framing of trust and trustworthiness. We regard regulatory sandboxes and RWT as trust domains because performative acts remain relevant to the evaluation of trust and trustworthiness, as are the associated values and norms that underscore the governance regime of the AI Act. However, these trust domains are ‘special’ in the sense that they have separate legislative treatment, as different proxies of trustworthiness and regulatory participation may be required. This in turn puts the focus on negotiations that are needed to put in place new proxies and accountability mechanisms to avoid fault finding or blame attribution that could hold back innovation.

### Regulatory Sandbox

The AI Act requires EU member states to singly or jointly ensure that at least one AI regulatory sandbox is established at the national level to facilitate the development and testing of innovative AI systems under strict regulatory oversight prior to granting market access or permitting their use (Article 57(1)). A regulatory sandbox is intended to ‘ensure a legal framework that promotes innovation, is future-proof and resilient to disruption’ (Recital 138) and should be focused on addressing legal uncertainties that confront current and prospective providers, thereby also facilitating evidence-based regulatory learning (Article 57(9(d))). Its objectives are not limited to fostering AI innovation through a controlled experimentation and testing environment, but also seek to facilitate regulatory learning with a view to future adaptations of the governance framework (Recital 139).

Just as a researcher must successfully deliver the research aims as entrusted in the research proposal or protocol in order to be trustworthy, a ‘sandbox plan’ sets out the performance of trustworthiness that is expected of prospective providers. Such a plan is defined as ‘a document agreed between the participating provider and the competent authority describing the objectives, conditions, timeframe, methodology and requirements for the activities carried out within the sandbox’ (Article 3(54)). Additionally, a regulatory sandbox should be designed and implemented in a way that facilitates cross-border cooperation between national competent authorities and with the AI Board and AI Office (Article 57(15)). Where appropriate, the national regulator implementing the regulatory sandbox is to provide guidance to current and/or prospective providers and to facilitate the involvement of other regulatory agencies (e.g. a personal data protection agency), including those supervising the protection of fundamental rights and other interested stakeholders in the AI ecosystem, such as research and experimentation labs and civil society organisations (Article 58(2(f))). Prior to authorising testing within a regulatory sandbox established under the AI Act, the national regulator must agree with all participants on the terms and conditions of the testing, particularly those that relate to appropriate safeguards to protect fundamental rights, health and safety (Article 58(4)). While providers remain liable for any damage inflicted on third parties as a result of experimentation within the sandbox, no administrative fines may be imposed under the AI Act if their participation was conducted in good faith and in accordance with specific plans and associated terms and conditions agreed upon with national regulator(s) (Article 57(12)).

Within the regulatory sandbox, personal data lawfully collected for other purposes may be processed solely for the purposes of developing, training and testing certain AI systems in the sandbox when all of the specified conditions are met (Article 59). Here, we set out those that are most pertinent to the context of healthcare and digital health:

- AI systems developed for safeguarding substantial public interest by a public authority or another natural or legal person governed by public law or by private law in one or more of the specified areas. This includes (as per Article 59(1)(a)(i)) public safety and public health, including disease detection, diagnosis prevention, control and treatment and improvement of health care systems.
- Data processes are necessary for complying with one or more of the requirements where those requirements cannot be effectively fulfilled by processing anonymised, synthetic or other non-personal data (see generally, Title III Chapter 2 of the AI Act).
- Effective monitoring mechanisms to identify if any high risks to the rights and freedoms of the data subjects, as referred to in Article 35 of Regulation (EU) 2016/679 and in Article 39 of Regulation (EU) 2018/1725 may arise during the sandbox experimentation as well as response mechanism to promptly mitigate those risks and, where necessary, stop the processing or use.
- Any personal data to be processed in the context of the sandbox are in a functionally separate, isolated and protected data processing environment under the control of the prospective provider, and only authorised persons have access to the data.
- Providers can only further share the originally collected data in compliance with EU data protection law. Any personal data in the sandbox cannot be shared outside the sandbox.
- Processing of personal data in the context of the sandbox do not lead to measures or decisions affecting the data subjects or the application of their rights in EU law on the protection of personal data.
- Any personal data processed in the context of the sandbox are protected by means of appropriate technical and organisational measures and deleted once the participation in the sandbox has terminated or the personal data has reached the end of its retention period.
- Logs of the processing of personal data in the context of the sandbox are kept for the duration of the participation in the sandbox, unless otherwise provided by EU or national law.
- Complete and detailed description of the process and rationale behind the training, testing and validation of the AI system is kept together with the testing results as part of the technical documentation.
- Short summary of the AI project developed in the sandbox, its objectives and expected results are published on the website of the competent authorities, but sensitive operational data in relation to the activities of law enforcement, border control, immigration or asylum authorities may be excluded.

Where activities may occur outside of the sandbox, existing legal obligations apply. For instance, the transfer of personal data will be subject to GDPR requirements, while appropriate safeguards for non-personal data may need to be put in place in accordance with the Data Governance Act (European Parliament and European Council 2022) and the Data Act (European Parliament and European Council 2023). Regulatory sandboxes are also intended to enable ‘interdisciplinary cooperation between AI developers, experts on inequality and non-discrimination, accessibility, consumer, environmental, and digital rights, as well as academics’ in order to achieve socially and environmentally beneficial outcomes (Recital 142), as well as provide support for small and medium size enterprises, including start-ups (Recital 143).

These are still early days of regulation on AI regulatory sandboxes, and more developments can be expected to follow. Under the AI Act, the European Commission is to adopt an implementation act specifying the modalities for the establishment, development, implementation, operation and supervision of the AI regulatory sandboxes. Common principles on eligibility and selection for participation in the regulatory sandbox, procedure for the application, participation, monitoring, exiting from and termination of regulatory sandbox and the terms and conditions applicable to participants are to be incorporated in the implementing act (Article 58(1)). What is clear at this stage is that new proxies of trustworthiness will need to be devised even if the values and norms identified by the AI HLEG continue to apply.

### Real-World Testing outside of a Regulatory Sandbox

High-risk AI systems may be tested in real world conditions outside AI regulatory sandboxes. In the healthcare and digital health contexts, such AI systems affect access to and enjoyment of essential private services and essential public services and benefits, particularly those used by or on behalf of public authorities to evaluate the eligibility of natural persons for essential public assistance benefits and services including healthcare services and AI systems intended to be used for risk assessment and pricing in relation to natural persons in the case of life and health insurance (Annex III, paragraph 5(a) and 5(ca)). Proxies of trustworthiness include the submission of a ‘real-world testing plan’, defined as ‘a document that describes the objectives, methodology, geographical, population and temporal scope, monitoring, organisation and conduct of testing in real-world conditions’ (Article 3(53)) and other performative acts specified therein. Performative acts of trustworthiness are not solely determined by a prospective provider as trustee since a national authority or regulator has the right to request information related to testing in real-world conditions from providers (through the power to conduct inspection for instance), require registration of the test in an EU-wide database, limit the duration of such testing, prescribe additional safeguards for persons belonging to certain vulnerable groups, require a clear definition of roles and responsibilities in a written agreement and impose additional safeguards designed specifically for testing in specific contextual applications like law enforcement and border control management (Article 60(4)).

In addition, the governance regime includes mechanisms that elicit acts of trustworthiness under certain conditions. For instance, providers are required to report any serious incident to the national regulator and to adopt immediate mitigation measures or otherwise suspend the testing, and must establish a procedure for the prompt recall of the AI system upon the termination of testing (Articles 60(7) and 60(8)). Information on serious incidents may also need to be shared under certain conditions. Providers are liable under applicable EU or a member state’s liability legislation for any damage caused in the course of their participation in testing in real world conditions (Article 60(9)). Remedies (more fully set out in section 4 of Chapter IX of the AI Act) may be exercised as a right to lodge a complaint with the national regulator (Article 85), a right to explanation of individual decision-making (Article 86), and reporting of breaches and protection of reporting persons (Article 87).

Certain features of RWT as a trust domain resemble those of ethical governance of research involving human participants. This may explain why only RWT of high-risk AI system may be required to undergo ethical review under national or EU law (Article 60(3)). One such feature is the prominent place that requirements relating to informed consent have as a regulatory safeguard in RWT (Article 61). Deployers or users of AI systems must be informed of all aspects of the testing that are relevant to their decision to participate, and be given instructions on how to use these systems (Article 62). However, informed consent may be waived if seeking consent would prevent the AI system from being tested, and only if the testing and outcome would not have any negative effect on the subject (Article 60(4)(i)). For informed consent to be legally valid, ‘concise, clear, relevant, and understandable information’ must be provided regarding (Article 61(1)):

- The nature and objectives of the testing and possible inconvenience that may be linked to their participation
- Conditions under which the testing in real world conditions is to be conducted (including expected duration of the subject’s participation)
- The participant’s rights and guarantees regarding participation (particularly right to refuse participation and to withdraw)
- The modalities for requesting the reversal or the disregard of the predictions, recommendations or decisions of the AI systems
- The EU-wide unique single identification number of the testing and contact details of the provider or its legal representative

Procedurally, informed consent is to be dated and documented, and a copy is to be provided to the subject or his or her legal representative (Article 61(2)).

Once the test is performed, the subject’s personal data is to be deleted, assuming that no subsequent validation is required. Testing must be overseen by the provider and deployer(s) with persons who are suitably qualified in the relevant field and have the necessary capacity, training, and authority to perform their tasks (Article 60(4)(j)). Any subject (or their legally designated representative) may withdraw from the testing at any time by revoking their informed consent and request the immediate and permanent deletion of their personal data, without any resulting detriment and without having to provide any justification. However, withdrawal of consent should not affect the activities already carried out (Article 60(5)). Where appropriate, safeguards could also include means to ensure that predictions, recommendations or decisions of AI system may be effectively reversed and disregarded, and that personal data may be deleted once consent has been withdrawn (Article 60(4(k))).

It seems to us that a regulatory sandbox and a RWT plan that is backed by law operate as trust domains within a wider epistemic environment of trust that the AI Act seeks to establish. Unlike the four-tier risk-based infrastructure that the legislation establishes, these trust domains apply to high-risk AI systems where new proxies of trustworthiness may need to be put in place. They allow for the goals of entrustment, the scope and boundaries of trust, and the order of performative acts to be negotiated through active engagement among the actors concerned. Within a regulatory sandbox, regulators can actively engage with providers as trustee on the boundaries and scope of personal data protection that must be sustained for the purposes of the tasks that are performed to achieve mutually agreed upon goals. New proxies of trustworthiness may be introduced as the socio-epistemic trust infrastructure evolves through regulatory learning. A RWT plan that is implemented as a trust domain under the AI Act operates in a similar manner, but it is likely to involve a wider range of participants, especially individuals that entrust their personal data to providers and deployers. From a governance perspective, a RWT plan has a number of features that are similar to those of a research proposal or protocol, in respect of which we have focused on the requirement of informed consent. Moving forward, we expect the requirements that national regulators issue collaboratively with the AI Office are likely to resemble those that apply to the ethical governance of research involving human participants. A hint to this is in the statutory provision stating that the RWT of high-risk AI systems may be required to undergo ethical review. If so, the analysis provided by Harvey and Laurie (2024) will be of especial relevance to thinking about trust and trustworthiness.

## Conclusion

In the digital health and healthcare contexts, we have considered how the EU seeks to create an epistemic environment of trust through the AI Act to facilitate the development and uptake of trustworthy AI systems. The legislation establishes a governance regime that operates as socio-epistemological infrastructure of trust which enables a performative framing of trust and trustworthiness. In this sense, we interpret trust and trustworthiness like Carter’s ‘trust as performance’ approach. The degree of success that performative acts of trust and trustworthiness have achieved in realising the legislative goals may then be assessed in terms of statutorily defined proxies of trustworthiness, as proposed by Harvey and Laurie. The assessment itself includes, as a component, the aptness of these performative acts. To be apt, these acts should be consistent with the ethical principles set out by the AI HLEG, as manifested in at least four key features of the governance regime. In other words, the proxies of trustworthiness make manifest the normative components of trustworthiness. Through the regulatory construct of risks, performative acts may be mandated and moderated through regulatory control which, as a matter of governance, implements an epistemic division of labour. This is most evident in the risk classification scheme that apply to AI systems that are not explicitly prohibited.

However, specified proxies of trustworthiness are not expected to be adequate for certain applications of AI systems, or would otherwise fall outside of the structured division of epistemic labour. Such applications are referred to as a regulatory sandbox and RWT of AI systems, which we consider to be ‘special’ trust domains for at least three reasons. First, the dynamic nature of these applications requires proxies of trustworthiness that are more responsive than those devised for the structured governance regime based on risk categorisation. Second, the governance regime (as socio-epistemological infrastructure of trust) is itself expected to evolve through regulatory learning. Third, these new and dynamic proxies of trustworthiness require active contributions from different stakeholders along the AI value chain, and it is in this sense ‘participatory’. For instance, proxies of trustworthiness in a sandbox plan and a RWT plan are likely to require some degree of negotiation and (after implementation) adaptation involving the trustor, trustee and regulatory intermediaries, rather than take the form of a reasonable offer from a researcher or research institution (as trustee) in a more classical health research setting.

Moving forward, more developments can be expected to follow from actions that are prescribed under the AI Act. A laissez-faire environment has contributed to important advancements in AI systems, but they have also raised public alarm that has fanned distrust in various quarters. Where the AI Act is concerned, there is a need to scale up from the current piecemeal and sporadic innovation of AI systems across the EU. To do so, different proxies of trustworthiness are required for an epistemic environment of trust aimed at realising social goals that are backed by appropriate values and norms, and legally sanctioned.

## Footnotes

1 Unless otherwise mentioned, all recitals and articles referred to in this paper are references to those of the AI Act (European Parliament 2024b).
